# Female Sports Medicine Fellowship–Trained Academic Orthopaedic Surgeons Earn Significantly Lower Salaries Than Their Male Counterparts

**DOI:** 10.1002/ars2.70013

**Published:** 2026-04-29

**Authors:** Udit Dave, Shreya M. Saraf, Mary K. Mulcahey

**Affiliations:** ^1^ Department of Orthopedic Surgery St. Lukes's University Health Network Bethlehem Pennsylvania U.S.A.; ^2^ Department of Orthopaedic Surgery and Rehabilitation Loyola University Medical Center Maywood Illinois U.S.A.

## Abstract

**Purpose:**

To identify whether there is a gender‐based disparity in salary among sports medicine fellowship–trained academic orthopaedic surgeons.

**Methods:**

Deidentified faculty compensation data were obtained from the Association of American Medical Colleges, which are compiled after distributing surveys to 157 Liaison Committee on Medical Education–accredited medical schools through a deidentified internet‐based survey application. Mean and median data for the 2023 calendar year was extracted from this dataset for male and female sports medicine fellowship–trained orthopaedic surgeons and stratified in a cross‐sectional fashion by position, including assistant professor, associate professor, and professor. Independent sample t‐tests and Cohen's *d* test were performed with Python and shown through a bar graph.

**Results:**

A total of 312 orthopaedic surgeons were included in this analysis, with 268 (85.9%) male and 44 (14.1%) female surgeons. Sports medicine fellowship–trained female surgeons earn significantly lower salaries than their male counterparts at the positions of assistant professor ($504,994 vs $654,697; *P* < .001), associate professor ($617,612 vs 776,754; *P* < .001), and professor ($486,303 vs $820,406; *P* < .001). The effect size of the difference between male and female salaries is greatest at the position of professor (*d* = 7.5), although there is a large difference in means between male and female assistant professors (*d* = 4.2) and associate professors (*d* = 3.4).

**Conclusions:**

Female sports medicine fellowship–trained academic orthopaedic surgeons earn significantly lower salaries at the assistant professor, associate professor, and professor levels. However, data were not able to be stratified based on additional variables that may influence salary among orthopaedic surgeons such as age, years in practice, geographic location, practice focus, and surgical volume.

**Clinical Relevance:**

This study shows that gender‐based disparities exist in compensation among sports medicine fellowship–trained academic orthopaedic surgeons.

## Graphical abstract

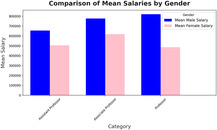

Orthopaedic surgery is a historically male‐dominated field, with female orthopaedic surgeons accounting for roughly 14% of all orthopaedic surgery residents.^1^, ^2^, ^3^ Although this proportion has been steadily increasing in recent years, there still exists a significant disparity in the number of female orthopaedic surgeons.^1^, ^2^, ^3^ In addition to a disparity in the number of orthopaedic surgeons who are female, there exists a discrepancy in salary earned by male and female orthopaedic surgeons.^4^, ^5^, ^6^, ^7^, ^8^ Prior literature has reported that male orthopaedic surgeons earn significantly more than female orthopaedic surgeons after controlling for case volume, with estimates for males’ annual income being around $800,000 versus females’ annual income being around $560,000.^4^, ^5^, ^6^ Furthermore, there exist inequities in consulting fees and royalties earned by male versus female orthopaedic surgeons.^7^ Previous studies have also shown salary inequity to lead to increased levels of burnout among female orthopaedic surgeons by contributing to feelings of devaluation.^9^, ^10^, ^11^

These overall trends observed across orthopaedic surgery are displayed within the subspeciality of sports medicine as well. In particular, only 14.4% of sports medicine fellows are female.^12^ Furthermore, 85.6% of sports medicine leadership roles such as program director or assistant program director are held by men.^12^ Previous studies have defined a gender‐based disparity in income across orthopaedic surgery and other subspecialties.^4^, ^5^, ^6^ The purpose of this study was to identify whether there is a gender‐based disparity in salary among sports medicine fellowship–trained academic orthopaedic surgeons. We hypothesized that female sports medicine fellowship–trained academic orthopaedic surgeons earn significantly less than their male counterparts.

## METHODS

### Dataset

This study was registered with the institutional review board and was granted exemption. Deidentified faculty compensation data were obtained from the Association of American Medical Colleges, which were compiled after distributing surveys to 157 Liaison Committee on Medical Education–accredited medical schools through a deidentified internet‐based survey application.^13^ Total salary was defined as a summation of fixed salary, earnings linked to medical practice revenue, and bonus or incentive pay from performance target metrics for which mean and median data were reported. Data for sports medicine orthopaedic surgeons were compiled independently; however, the aforementioned components of the total salary were not individually reported. Salary data for the 2023 calendar year were extracted from this dataset for male and female sports medicine fellowship–trained orthopaedic surgeons and stratified in a cross‐sectional fashion by position, including assistant professor, associate professor, and professor. Raw data were not available for 2023 for the positions of instructor, chief, and chair; therefore, these positions were excluded from our analysis. Data points reported included mean salary, median salary, and total number of male and female surgeons at each position.^13^

### Statistical Analysis

Although the Association of American Medical Colleges dataset reported mean and median, the dataset was limited in that it did not report a measure of spread such as a standard deviation (SD) or interquartile range, and raw salary data were unavailable to perform these calculations. Therefore, SD for each group was estimated by using the relative standard error (RSE) of all orthopaedic surgeon salaries for 2023 reported by the United States Bureau of Labor Statistics.^14^ These SDs were then used to perform an independent sample t‐test to evaluate for a statistically significant difference in salary between male and female individuals at each position, reported as a *P* value. Additionally, a Cohen's *d* statistic was calculated to evaluate the magnitude of the difference in salary between male and female individuals at each position. A standard alpha value of 0.05 was set for all tests of statistical significance. A bar graph was then produced to summarize salaries for male and female individuals at each position. All statistical analysis and graph creation was performed using the software Python (Python Software Foundation, Beaverton, OR).

## RESULTS

### Demographics

A total of 312 orthopaedic surgeons were included in this analysis, with 268 (85.9%) male and 44 (14.1%) female surgeons. There were a total of 154 assistant professors, 87 associate professors, and 71 professors included in this study. Among the assistant professors, 129 (83.8%) were male and 25 (16.2%) were female. Among the associate professors, 75 (86.2%) were male and 12 (13.8%) were female. Among the professors, 64 (90.1%) were male and 7 (9.9%) were female.

### Salary

The RSE of orthopaedic surgeon salaries reported by the US Bureau of Labor Statistics for 2023 is 5.6%. Given that RSE = SD/mean, the SD for each salary group was estimated by multiplying the mean salary by 0.056. Female assistant professors earned a mean of $504,994 versus $654,697 (*P* < .001, *d* = 4.2) for male surgeons. Additionally, female associate professors earned a mean of $617,672 versus $776,754 (*P* < .001, *d* = 3.7) for male surgeons. Lastly, female professors earned a mean of $486,303 versus $820,406 (*P* < .001, *d* = 7.5) for male surgeons.

**FIGURE 1:**
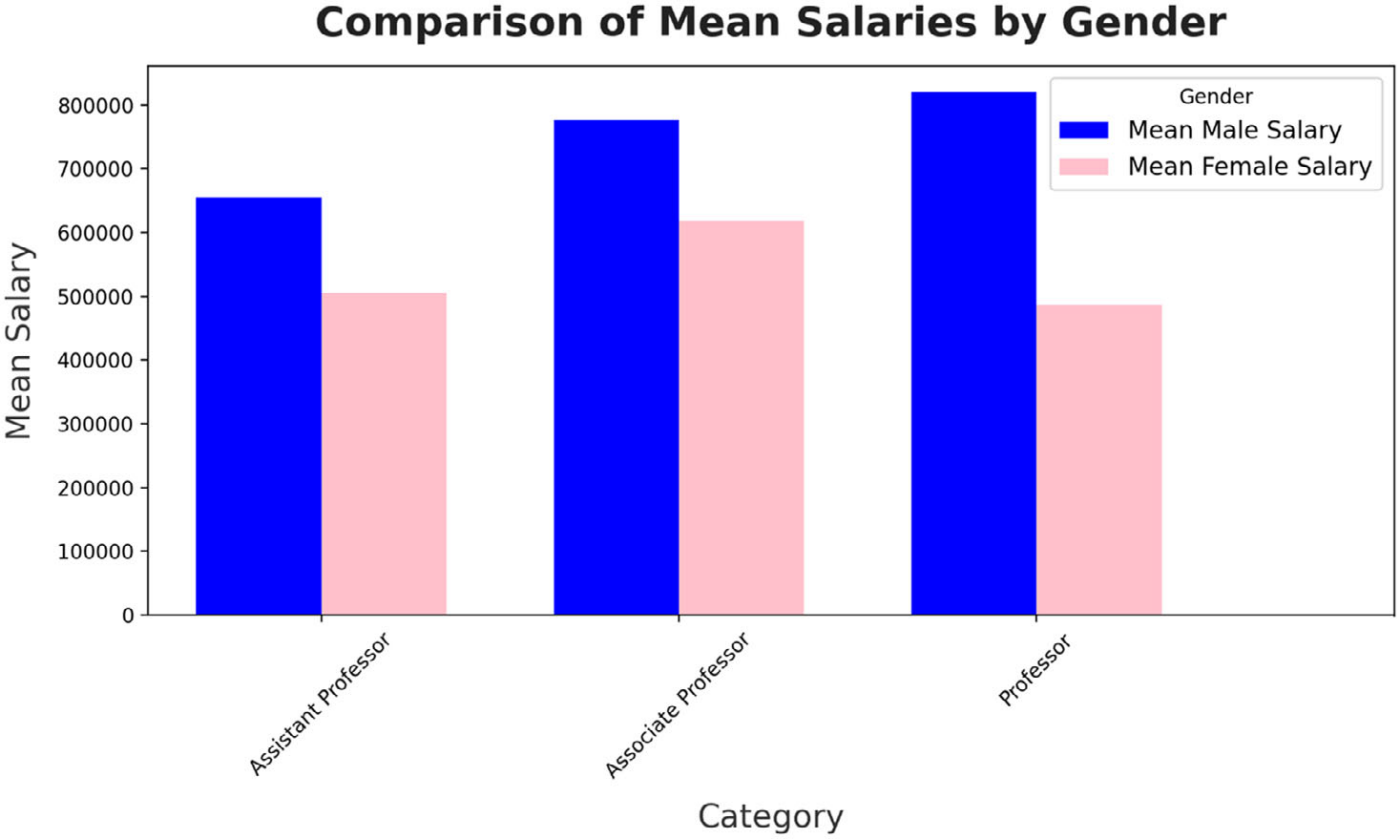
Bar graph displaying salary for male and female orthopaedic sports medicine surgeons at the positions of assistant professor, associate professor, and professor.

Female orthopaedic sports medicine fellowship–trained surgeons earned significantly lower salaries than their male counterparts at each position as measured by independent t‐test. Furthermore, there is a large effect size in difference in salary at each position. In particular, the largest magnitude of difference as measured by Cohen's coefficient is seen at the level of professor. Table 1 provides a summary of all demographics and statistical analyses performed comparing salaries of female and male surgeons at each position. Figure 1 displays a bar graph illustrating the salaries of male and female surgeons at each position.

**TABLE 1:** Summary of Salary Differences by Gender

Position	N Males	Mean Male Salary (SD) in $	N Females	Mean Female Salary (SD) in $	Cohen's *d* (Effect)	*P* Value	Conclusion
Assistant professor	129	654,697 (36,663)	25	504,994 (28.280)	4.2 (large)	*P* < .001	Significant
Associate professor	75	776,754 (43,498)	12	617,612 (34,591)	3.4 (large)	*P* < .001	Significant
Professor	62	820,406 (45,943)	7	486,303 (27,233)	7.5 (large)	*P* < .001	Significant

## DISCUSSION

The most significant finding of this study is that female orthopaedic sports medicine fellowship–trained surgeons earn significantly lower salaries than their male counterparts at the positions of assistant professor ($504,994 vs $654,697; *P* < .001), associate professor ($617,612 vs 776,754; *P* < .001), and professor ($486,303 vs $820,406; *P* < .001).

Of note, the greatest difference in salary was observed among professors, where male professors earned more than $300,000 more than female professors. Interestingly, mean salary among female associate professors was greater than mean salary among professors. A reduction in mean salary despite a higher position could be explained by a decrease in case load or hours worked; however, previous studies have established that there is no difference between male and female orthopaedic surgeons with regard to leadership goals, research endeavors, or projected work schedule.^4^, ^15^ These previous findings suggest that although there is a paradoxical decrease in salary at a higher position among female orthopaedic surgeons included in this survey that may be explained by decrease in case load, it is likely not indicative of a fundamental difference with regard to gender in leadership goals. An overall disparity in salary between male and female orthopaedic surgeons, regardless of subspecialty, was reported by Bebee et al. (mean difference $62,032.51), Jena et al. (mean difference $40,953, 95% confidence interval $2,277 to $79,628), and Ponzio et al. (mean difference $89,000, *P* < .001).^4^, ^16^, ^17^

Prior literature has also established that after accounting for seniority and level of training, female orthopaedic surgeons receive fewer referrals than their male counterparts and that female surgeons receive overall lower Medicare reimbursements.^18^ These data were evaluated by comparing reimbursement codes, and the authors suggested that these disparities existed due to female surgeons being more likely to adhere to clinical guidelines and on average spending more time with each of their patients.^18^ Identifying differences in income among sports medicine fellowship–trained orthopaedic surgeons is critical in order to continue addressing gender‐based inequities within the subspecialty. We also found drastic differences regarding leadership roles held by male versus female orthopaedic surgeons. In our study, the greatest effect size in difference in mean salary was observed at the professor level. There were only 7 female professors included within our study compared with 62 male professors, which is indicative of an inherent disparity in attaining leadership positions within sports medicine. Previous studies have reported that across orthopaedic surgery, female individuals serve in significantly fewer leadership positions, making up a smaller proportion of residency program directors than every specialty except for neurosurgery.^2^, ^5^, ^17^, ^19^ The disparity in leadership positions held by female individuals in orthopaedic surgery may partially be due to the younger overall age of female individuals in the specialty as 16% of orthopaedic surgeons younger than 40 years of age are female compared with 8% of surgeons in their 40s being female and 7% of surgeons in their 50s being female.^20^ However, age only paints part of the picture as previous studies have shown that female individuals in academic medicine are commonly affected by anxiety stemming from stereotype threat, a phenomenon in which negative stereotypes affect performance in a group setting.^20^, ^21^ Our findings show that the numerous factors that have led to a gender‐based disparity in leadership positions within orthopaedic surgery as a whole are also at play within the sports medicine subspecialty.

Our study has several strengths. We were able to determine a gender‐based difference in income for fellowship‐trained sports medicine surgeons while stratifying for level of academic position. Furthermore, we were able to determine effect size in addition to statistical significance. Further primary studies are required that directly compare income specifically among fellowship‐trained sports medicine surgeons that account for factors such as case volume and scope of practice. Additionally, future studies should investigate male and female sports medicine surgeons’ attitudes toward negotiating salaries to evaluate whether there exists a disparity on the basis of gender.

### Limitations

There are several limitations to this study. This study only evaluates sports medicine orthopaedic surgeons in an academic context; therefore, we are unable to evaluate potential gender‐based disparities in salary in a private practice context. The dataset used in this study was compiled by the Association of American Medical Colleges after distributing surveys to 157 Liaison Committee on Medical Education–accredited medical surveys. Response bias is an inherent limitation of any survey‐based study, especially one requesting individuals to provide sensitive personal information such as their salary. Furthermore, we were unable to stratify these data based on additional variables that may influence salary among orthopaedic surgeons such as age, years in practice, geographic location, practice focus, and surgical volume. Additionally, a major limitation of this dataset was that SD data for salary were not provided. The SDs used were estimated by using the RSE of orthopaedic surgeon salaries for 2023 reported by the United States Bureau of Labor Statistics; however, these data are not specific to sports medicine fellowship–trained orthopaedic surgeons and potentially underestimate or overestimate the sports medicine surgeon–specific SD.

## CONCLUSIONS

Female sports medicine fellowship–trained academic orthopaedic surgeons earn significantly lower salaries at the assistant professor, associate professor, and professor levels. However, data were not able to be stratified based on additional variables that may influence salary among orthopaedic surgeons such as age, years in practice, geographic location, practice focus, and surgical volume.

## DISCLOSURES

The author (M.K.M.) declares the following financial interests/personal relationships, which may be considered as potential competing interests: M.K.M. reports board membership with the American Association of Orthopaedic Surgeons, the *American Journal of Sports Medicine*, the American Orthopaedic Association, the American Orthopaedic Society for Sports Medicine, *Arthroscopy*, the Arthroscopy Association of North America, the Association of Bone and Joint Surgeons, the International Society of Arthroscopy, Knee Surgery and Orthopaedic Sports Medicine, and the Ruth Jackson Orthopaedic Society; consulting or advisory and speaking and lecture fees from Arthrex Inc. The other authors (U.D., S.M.S.) declare that they have no known competing financial interests or personal relationships that could have appeared to influence the work reported in this article.
